# Medical students' and faculty members' perceptions and experiences of AI integration in health care practice and in medical curricula: A meta‐ethnographic review

**DOI:** 10.1111/medu.70071

**Published:** 2025-11-05

**Authors:** See Chai Carol Chan, Holly Young, Ravi Parekh

**Affiliations:** ^1^ Department of Primary Care and Public Health, School of Public Health Imperial College London London UK; ^2^ Centre for International Medical Education Collaborations UCL London UK

## Abstract

With the increasing adoption of artificial intelligence (AI), health care systems and medical education are undergoing significant changes. This review examines how medical students and faculty members perceive the opportunities and challenges of AI integration in both health care practice and medical curricula.

A meta‐ethnographic approach, following the eMERGe guidelines, was used to synthesise qualitative research that focuses on perceptions and experiences among students and faculty members. Systematic searches were conducted across ERIC, Embase, PsycINFO, Web of Science and Medline databases, resulting in 1087 articles. Following an assessment of methodological robustness, 26 articles that met the inclusion criteria were included.

The synthesis incorporated insights from 4380 students and 75 faculty members from at least 48 countries. There were differing experiences and perceptions of AI in health care and its integration in medical curricula. Four third‐order constructs were developed. “Implications on clinical practice” demonstrates how these participants view AI as a decision support tool and its impact on humanistic relationships and efficiency. “AI integrity” considers their perspectives on trust, accountability, inequity and the ethical use of AI technologies. “Educational implications and preparedness” examines preparation for the future workforce and approaches and barriers to integration in medical curricula. “Future workforce” considers participants' perspectives related to the evolving roles of health care professionals in an AI‐driven landscape.

This review discusses the complex interactions between AI integration in health care practice and in medical curricula, revealing challenges and opportunities as perceived by students and faculty members. Although AI has the potential to revolutionise health care practices, significant educational gaps still hinder its effective implementation. This review advocates for curricula to better tailor to the specific needs of students and faculty members. It also emphasises the importance of incorporating ethical considerations and cross‐disciplinary collaboration to ensure readiness for an AI‐driven future in health care.

## Abstract 2

AI is transforming medicine—but are global medical education systems ready? Our review examines how students and faculty perceive AI integration and what needs to change in medical education. #AIinMedicine #MedEd

## 1 INTRODUCTION

### 1.1 Artificial intelligence in health care practice

Artificial intelligence (AI) refers to the ability of computers to perform tasks that traditionally require human intelligence.^1^ AI has widespread applications in health care, including disease prediction,^2^ chronic disease monitoring,^3^ clinical diagnostics^4^ and improving documentation and efficiency in care management.^5^ Throughout this review, we adopt the term “AI in healthcare practice” to encompass this wide range of such applications. Despite its potential, AI integration presents complex challenges, including concerns about data privacy, security, transparency and algorithmic bias.^6^, ^7^ Furthermore, the ‘black box’ nature of many AI systems obscures their internal workings from clinicians, patients and regulators.^8^ Therefore, not only is it difficult to validate AI‐driven decisions, but it also hinders the transparency needed for trust and accountability in health care settings.^6^, ^9^ Another challenge is algorithmic bias. When AI models are trained on datasets that are not representative of the target population,^10^ it can lead to discriminatory diagnosis, biased treatment decisions and exacerbate existing health inequities.^11^ Addressing these challenges requires stringent protocol development and deployment to ensure equitable and effective health care practices.^12^

Recognising these issues, the World Health Organisation^13^ has emphasised the importance of inclusivity, transparency and accountability in AI applications within health care in its published guidance. In response, instruments, such as the Quality Assessment of Diagnostic Accuracy Studies (QUADAS‐AI) and Transparent Reporting of a multivariable prediction model for Individual Prognosis Or Diagnosis (TRIPOD), have been established to help researchers and policymakers evaluate the risk of bias and applicability in AI tools.^14^, ^15^ Ensuring these technologies are ethical and effective in improving health care outcomes requires comprehensive collaboration.^12^ Health care professionals, policymakers, patients and AI developers need to actively engage with one another across every phase of AI development: from problem identification and data collection to implementation and post‐deployment evaluation.^11^, ^12^

### 1.2 Artificial intelligence in medical curricula

Over the past decade, particularly accelerated by the COVID‐19 pandemic, medical education has increasingly integrated more digital and AI components into its curricula.^7^ This shift aims to enhance students' understanding of AI application in health care settings and equip them with skills to effectively use and critique these technologies.^2^

However, the extent of AI integration in medical education varies significantly. Although some universities in high‐income countries are actively integrating AI topics into their medical curricula,^16^, ^17^, ^18^ medical schools in lower‐income regions face barriers such as limited access to advanced technology and infrastructure.^19^, ^20^, ^21^, ^22^

Beyond institutional and geographical disparities, several common challenges hinder AI integration in medical curricula.^19^ Firstly, dense medical curricula leave little room to introduce new topics without overburdening students.^23^, ^24^ Secondly, a lack of faculty expertise limits the innovative educational strategies and teaching methods.^21^, ^25^ Lastly, accreditation standards have yet to recognise AI as a core component in medical education, meaning it remains absent from national examinations such as the United States Medical Licensing Exam.^16^

### 1.3 Study scope and rationale

Despite several reviews on AI integration in medical education,^1^, ^2^, ^25^, ^26^ there is a lack of systemic exploration of medical students' and faculty members' experiences and perceptions of AI integration in health care practice and medical curricula. Understanding these perspectives is crucial for advocating change through an inclusive, bottom‐up approach. This review aims to identify their perceived gaps and barriers relating to AI in medical education that may hinder students' preparedness for future practice. These insights will guide educators and policymakers with suggestions and strategies to revise the curricula.

Importantly, the focus of this review is on the inclusion of AI as a substantive subject within medical curricula, rather than its use as an educational tool, such as employing natural language processing or large language models (e.g. Chat‐GPT) for generating assessment feedback or creating simulated patient interactions.^27^, ^28^

### 1.4 Research aim

To explore medical students' and faculty members' perceptions and experiences of AI integration in health care practice and in medical curricula.

### 1.5 Positionality and reflexivity

As academic GP Registrars in the United Kingdom, SCCC and HY have taught and assessed students from different universities. Alongside clinical practice, they have academic interests in digital health, artificial intelligence and medical education. SCCC was raised in Hong Kong and had educational and professional experiences in the United Kingdom, providing her with a unique perspective on how Western and Non‐Western cultural nuances influence teaching and learning. Both SCCC and HY have engaged with AI in research and practice and hold an optimistic view of its integration in health care and in the medical curriculum. However, they acknowledge the ethical and practical challenges associated with AI and advocate for a balanced approach that ensures it complements rather than replaces clinical reasoning and human interaction.

As an academic GP, RP has led courses, taught, assessed and led a research centre at a UK higher education institution. His dual role in clinical and academic practice allows him to understand the impact of AI on educational curricula and patient care. He is not an expert in AI, but uses it for day‐to‐day tasks and remains cautiously optimistic about its role in health care and education.

## 2 METHODS

A meta‐ethnographic approach was used to systematically select, analyse and synthesise relevant qualitative literature. This method emphasises inductive reasoning and interpretation, aligning closely with the qualitative nature of the studies it synthesises.^29^ Meta‐ethnography also adds breadth and depth to existing systematic reviews by reducing duplication and exploring the relationship between individual experiences and broader systemic influences.^29^ Meta‐ethnography is widely used in Health Professions Education research to understand perceptions and experiences of different stakeholders.^30^, ^31^, ^32^

This review adheres to the eMERGe guidelines^33^ to ensure methodological transparency and rigour at each stage of the process.

### 2.1 Literature search strategy

The Population, Exposure, Outcome (PEO) method was adopted, which is a structured search strategy designed to target specific population groups, exposures and defined outcomes.^34^ The complete list of search terms and strategies is detailed in Appendix 1. The search approach was comprehensive rather than purposeful to ensure all available studies were included.

### 2.2 Inclusion and exclusion criteria

The inclusion and exclusion criteria are outlined in Table 1. This table specifies criteria on methodology, participant group, topic, language, source, publication date and geographical region.

**TABLE 1:** Inclusion and exclusion criteria.

Criteria type	Inclusion detail	Exclusion detail
*Methodology*	Studies employing qualitative methodologies (including mixed‐method research which have qualitative component).	Systematic reviews, meta‐analyses, opinion papers, editorials, commentaries and purely quantitative studies. Excluded if lacking participant verbatim quotes or analysis/ discussion of responses.
*Participants*	Undergraduate medical students and/or faculty members (including but not limited to doctors, residents, trainees and postgraduate students).	Health care students and professionals without formal teaching roles. Excluded if participant quotes are not clearly attributed to medical students or faculty members.
*Topic*	Focus on AI integration in health care contexts and/or in medical curricula.	Excluded if addressing digital health or telemedicine without AI being the central topic. Excluded if evaluating AI only as an educational tool.
*Language*	Articles published in English.	Excluded if not published in English.
*Source*	Peer‐reviewed journal articles.	Non‐peer‐reviewed articles, unpublished manuscripts, conference abstracts.
*Publication Date*	Articles from 2014 onwards, as they are more likely to reflect AI capabilities and applications.	Prior to 2014.
*Geography*	Global	N/A

### 2.3 Search results

**FIGURE 1:**
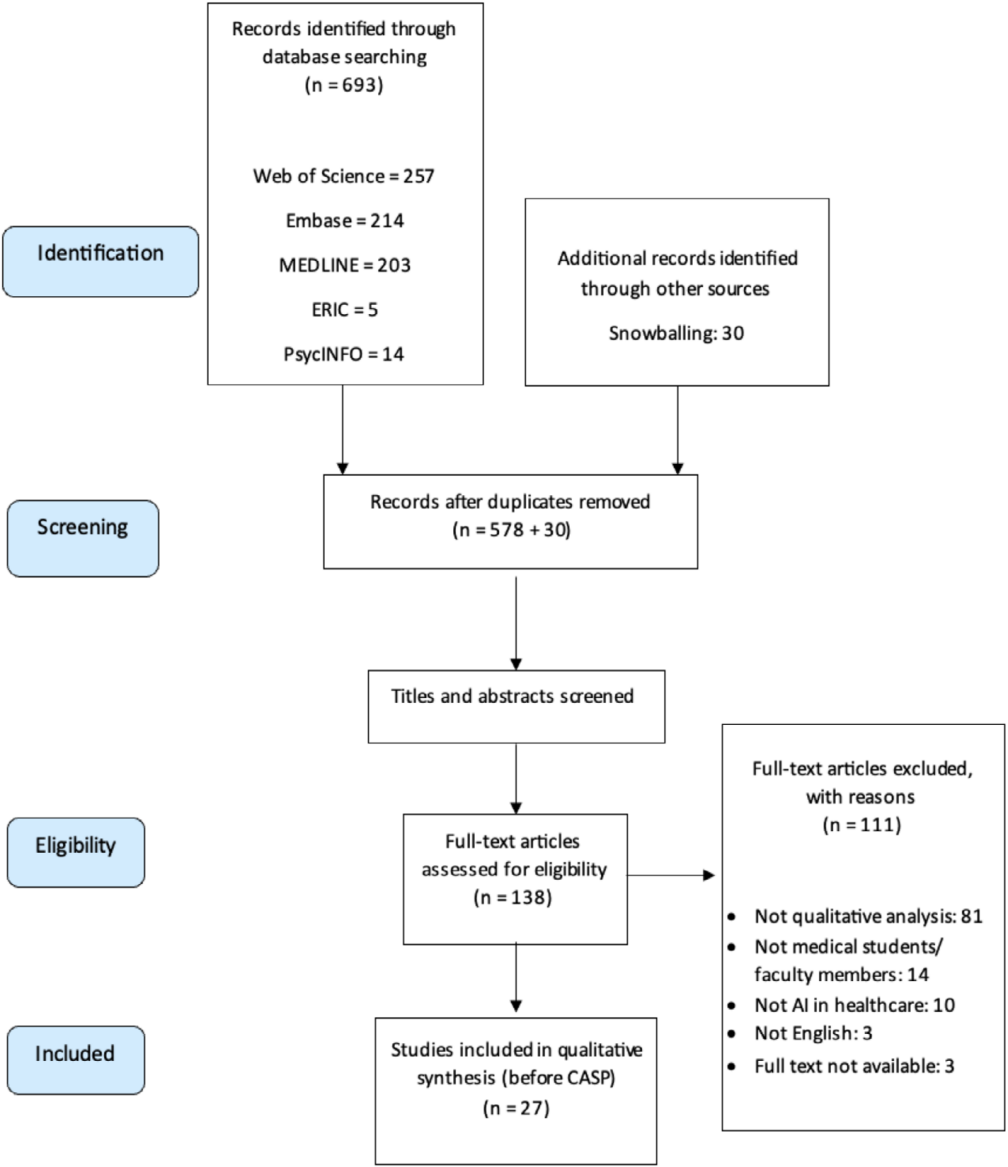
PRISMA diagram. [Color figure can be viewed at wileyonlinelibrary.com]

The search results from December 2024 are summarised in the Preferred Reporting Items for Systematic Reviews and Meta‐Analyses (PRISMA) diagram (Figure 1). After the removal of duplicate records, 578 records remained. Thirty potential articles were identified through snowballing techniques, including forward and backward citation tracking. Title and abstract screening narrowed the selection to 138 articles. Following full‐text review by SCCC and HY, 27 papers were deemed suitable for analysis.

### 2.4 Assessment of study quality and relevance

To ensure rigour and transparency, all included articles were appraised using the Critical Appraisal Skills Programme (CASP) qualitative research checklist.^35^ Studies with a CASP percentage score of 60% or higher were deemed of suitable quality for inclusion.

As outlined by Dixon‐Woods et al,^36^ articles were also classified as either a ‘Key Paper’ (KEY), where the content closely aligned with the research question, or a ‘Satisfactory Paper’ (SAT), where the content contributed less towards the synthesis.

### 2.5 Meta‐ethnographic synthesis

The data synthesis processes involved extracting direct quotes from each study to provide raw insights into the experiences of medical students and faculty members regarding AI integration in health care practice and in medical curricula. These quotes are known as first‐order constructs and served as the empirical foundation for the original study authors to derive second‐order constructs. These second‐order constructs offer thematic and theoretical understandings that contextualise individual experiences with broader academic discussions.^29^, ^33^

Third‐order constructs were then developed by comparing and translating second‐order constructs across studies. This process identified emergent and overarching themes that accounted for both similarities and discrepancies, following the line‐of‐argument synthesis approach.^29^, ^33^ This method enables new interpretations that transcend findings from individual studies to comprehensively answer the research question.^37^, ^38^ As noted by Campbell et al,^39^ this approach maintains academic rigour while allowing flexibility to accommodate diverse institutional contexts and geographical disparities.

## 3 RESULTS

### 3.1 Study quality and relevance

The methodological robustness varied amongst the 27 studies. One article was excluded due to poor methodological and ethical considerations. The remaining 26 studies demonstrated clear objectives, appropriate data collection methodologies and clear statements of findings. CASP percentage scores for these 26 studies ranged from 60% to 95% (Table 2).

**TABLE 2:** Characteristics of articles included in the meta‐ethnography.

First author, year	Sample population (year group)^1^	Country/region	Study design	Relevance^2^	CASP score^3^	Reference
Abid, 2024	4 students (fourth year)	United States	Survey	SAT	15/10	^40^
Ahmad, 2024	417 students	India	Survey	SAT	14/10	^41^
Alrashed, 2024	13 students	Saudi Arabia	Semi‐structured interviews Focus group discussions	SAT	18/20	^42^
Blease, 2022	153 students (final year)	Ireland	Survey	SAT	17/20	^43^
Busch, 2024	6 students	Nigeria, Switzerland, Germany, Canada, Portugal	Survey	SAT	16/10	^20^
Ejaz, 2022	128 students	48 countries in Africa, Asia, Oceania, Europe, South America and North America^4^	Focus group discussions Survey	SAT	18/20	^44^
Funer, 2024	15 students	Germany	Semi‐structured interviews	SAT	19/20	^45^
Funer, 2024	15 students	Germany	Semi‐structured interviews	SAT	18/20	^46^
Gandhi, 2024	605 students	India	Semi‐structured interviews Focus groups Survey	SAT	18/20	^47^
Gillissen, 2022	28 students	Germany	Semi‐structured interview Survey	SAT	16/20	^48^
Goetz, 2020	8 (5 first year, 3 fourth year)	United States	Focus groups	SAT	17/20	^49^
Gong, 2019	322 students	Canada	Survey	SAT	14/20	^50^
Jebreen, 2024	15 students	Palestine	Semi‐structured interviewsSurvey	Key	18/20	^22^
Jha, 2022	216 students	Nepal	Survey	SAT	14/20	^19^
Jussupow, 2022	164 students	Germany	Survey	SAT	16/20	^51^
Liu, 2022	390 students	United States	Survey	SAT	16/20	^23^
Mehta, 2019	321 students	Canada	Survey	SAT	17/20	^52^
Memon, 2023	20 faculty members	Pakistan	Semi‐structured interviews Survey	SAT	15/20	^21^
Moldt, 2023	12 students	Germany	Content analysis	SAT	17/20	^53^
Moldt, 2024	10 students, 6 faculty members	Germany	Semi‐structured interviews	SAT	17/20	^54^
Park, 2021	156 students	United States	Survey	SAT	12/20	^55^
Pucchio, 2022	486 students	Canada	Semi‐structured interviews Survey	SAT	19/20	^56^
Robleto, 2024	73 students	United States	Survey	SAT	16/20	^8^
Stewart, 2023	134 students	Australia	Survey	SAT	18/20	^57^
Shimizu, 2023	6 students, 49 faculty members	Japan	Content analysis	SAT	15/20	^58^
Teng, 2022	683 students	Canada	Survey	SAT	18/20	^24^

^**1**^ If the number of participants in the qualitative component of a study is not specified, the total study population is assumed.

^**2**^ Relevance was assessed using the Dixon‐Woods et al. criteria^36^; KEY denotes a Key Paper, whereas SAT represents a Satisfactory Paper.

^**3**^ CASP Score.^35^

^**4**^ The study did not specify which 48 countries the students represented.

Using the Dixon‐Woods et al criteria,^36^ one article was identified as ‘Key’ (Table 2). Jebreen et al^22^ focused on the same research questions as this review and employed a predominantly qualitative approach with in‐depth data analysis. The remaining 25 papers were classified as ‘Satisfactory.’ Most of these studies offered insights of potential interest, but relied on mixed‐method approaches that did not predominantly focus on qualitative data analysis.

### 3.2 Study characteristics

This review synthesised findings from 26 papers, involving 4380 students 1 and 75 faculty members. Study characteristics are detailed in Table 2. However, demographic details, such as students' year groups and faculty members' specialties, were inconsistently reported, limiting the ability to fully assess sample diversity and representation across the studies.

The geographical spread of the included studies was vast, covering at least 48 countries 2 across 7 regions: Europe, Middle East, Asia, North America, South America, Africa and Oceania. Despite the inclusion criteria spanning from 2014 to 2024, all included studies were published between 2019 and 2024, reflecting a growing interest and adoption of AI in health care and medical curricula.

The methodological approaches of the included studies varied. Eighteen studies used mixed‐methods surveys that included open‐ended questions, whereas others employed interviews, content analysis and focus groups.

In terms of content focus, some studies examined general AI use in health care.^19^, ^21^, ^56^ Other studies explored the impact of AI on specific careers, such as primary care^43^ and radiology.^50^ Some studies examined specific AI technologies, including virtual doctors^49^ and AI‐assisted algorithms.^8^

### 3.3 Third‐order constructs

Twelve second‐order constructs were identified and are detailed in Table 3, along with the articles from which they arise and representative first‐order constructs. These second‐order constructs were synthesised by the research team into four third‐order constructs:

- Implications on clinical practice
- AI integrity
- Educational implications and preparedness
- Future workforce

**TABLE 3:** Table collating the formulated third‐order constructs by researchers based on second‐order constructs extracted from research articles.

*Third order construct*	*Related second order construct*	Illustrative first order construct	Articles contributing to the second order construct
*Implications on clinical practice*	*Efficiency*	“...healthcare system could be significantly improved and streamlined with the use of AI‐assisted diagnostic tools.”^8^ “The paperwork burden of writing medical certificates and charts will be reduced.”^58^	^8^, ^19^, ^24^, ^41^, ^43^, ^47^, ^49^, ^51^, ^54^, ^57^, ^58^
	*Decision support tool*	“…it could help expedite diagnoses and help physicians consider every possible outcome when dealing with patients.”^8^ “AI is a relief for the physician and helps to gain security in diagnosing by providing a second opinion which either encourages the physician to reflect his diagnosis a second time or strengthens the certainty of having found the right diagnosis.”^51^	^8^, ^20^, ^22^, ^23^, ^41^, ^42^, ^43^, ^44^, ^45^, ^46^, ^47^, ^49^, ^50^, ^51^, ^52^, ^54^, ^55^, ^57^
	*Humanistic relationship*	“People want people to treat them.”^52^ “Doctor will change to be more of an educator and communicator.”^57^ “Empathy but also tactfulness is so important which can never be accomplished by a robot.”^48^ “The complete lack of touch, the complete lack of a physical exam would bother me.”^49^ “In terms of diagnosis, medicine is as much an art as a science. I find it difficult to believe that a computer can appreciate the value of a clinical decision based on observation.”^43^	^20^, ^22^, ^41^, ^43^, ^45^, ^47^, ^48^, ^49^, ^51^, ^52^, ^53^, ^57^, ^58^
*AI integrity*	*Ethics*	“How can we use AI to do good, and not harm, as a tool?”^44^ “Major ethical and legal issues will arise if (and when) mistakes are made using AI as a guide.”^57^ “Patient privacy and confidentiality concerns would need to be considered when using these tools.”^8^	^8^, ^21^, ^22^, ^23^, ^41^, ^42^, ^44^, ^47^, ^49^, ^53^, ^56^, ^57^
	*Trust and accountability*	“Liability, privacy and control over data and black box phenomena are the real challenges of using artificial intelligence in healthcare system.”^21^ “To what extent can I trust the AI, the outcomes it produces? How can I collaborate effectively with it? What do I need to operate a good AI, and where can it also be deployed?”^54^ “There is a need for transparent and rigorous validation of these tools to ensure their safety and reliability in clinical settings, as erroneous diagnoses or treatment recommendations can have serious consequences for patients.”^8^ “There is almost no governance over AI and no one seems to care.”^57^ “I would trust it very well, probably also more than people who operate without this assistance.”^46^	^8^, ^20^, ^21^, ^22^, ^23^, ^41^, ^45^, ^46^, ^48^, ^49^, ^51^, ^52^, ^53^, ^54^, ^57^
	*Inequity*	“There are deep inequalities and inequities…that have longstanding historical underpinnings, which AI will ignore and this is dangerous…”^52^ “Firstly, bias in AI algorithms can lead to disparities in healthcare outcomes if not properly addressed, as these tools may perform differently on diverse patient populations.”^8^ “… there are certain stigma or certain assumptions people make based on how they look… You don't have to deal with that when you're dealing with a tool.”^49^ “None of the medical college in Pakistan is working on artificial intelligence.”^21^ “Low‐ and middle‐income countries should have access to technology first … Some regions still don't have access to internet.”^44^ “Won't AI be for just the rich”^20^	^8^, ^19^, ^20^, ^21^, ^22^, ^23^, ^41^, ^44^, ^47^, ^49^, ^52^
*Educational implications and preparedness*	*Approaches to integration*	“Reducing lectures and increasing skills training and clinical clerkships.”^58^ “It should be more integrated into our curriculum instead of being viewed as a distant topic… since we are all going to be impacted by it.”^52^	^21^, ^22^, ^23^, ^40^, ^42^, ^44^, ^47^, ^50^, ^52^, ^54^, ^56^, ^57^, ^58^
	*Preparation for future workforce*	“…is important because I think that it's going to be a reality in how a physician practices medicine and should be something we should learn.”^56^ “I'm more than willing to learn to work with AI.”^57^ “This means that I will waste time studying topics that have no practical application in my country.”^22^	^19^, ^20^, ^22^, ^42^, ^44^, ^45^, ^46^, ^47^, ^48^, ^50^, ^56^
	*Barriers to integration*	“I think that… my time would be better spent understanding the body and having [AI tools and results] interpreted for me by somebody who is an expert.”^56^ “I feel that it's more important to learn about AI in residency versus medical school.”^23^ “I don't really care because I don't understand how it applies to me.”^24^ “I don't think medical students have enough computer science and engineering background to learn much about AI.”^23^	^21^, ^22^, ^23^, ^24^, ^52^, ^56^
*Future Workforce*	*Job security*	Physicians will be replaced by computers and will only fulfil an assistant job.^51^ “Artificial intelligence won't replace doctors. Instead, doctors who can use artificial intelligence will replace doctors who can't use it.”^57^ “There will likely be a transition period of physician‐monitored use of AI tools, or AI‐assistance of physician tasks, rather than a complete replacement.”^52^	^19^, ^20^, ^22^, ^23^, ^40^, ^41^, ^43^, ^47^, ^48^, ^49^, ^50^, ^51^, ^52^, ^55^, ^57^, ^58^
	*Emotions*	“It does concern me however that I have spent a long time studying and potentially will not have a job that I worked hard for.”^57^ “I feel we, as students, are confused about our future role when AI is introduced.“^20^ “It is simply not our field and expertise and it stresses me out.”^24^ “I'm not afraid of change and I'm more than willing to learn to work with AI.”^57^	^20^, ^22^, ^23^, ^24^, ^47^, ^50^, ^57^
	*Specialty*	“AI could potentially make radiologist(s) obsolete”^55^ “I am interested in a field of medicine (internal medicine /physician) which I don't think artificial intelligence will ‘replace’ over my lifetime.”^57^ “Maybe there'll be less jobs for physicians in certain fields that AI is more applicable to, like radiology or pathology.”^56^ “AI will be more applicable than human doctors, such as pathology, radiology, cardiology, dermatology, and ophthalmology.”^22^	^19^, ^22^, ^40^, ^43^, ^49^, ^50^, ^55^, ^56^, ^57^

#### 3.3.1 Implications on clinical practice

AI technologies have improved efficiency and operations across health care, including patient scheduling and record management.^44^ The second‐order constructs highlight AI's dual role as a facilitator and disruptor of clinical practice. Although some participants perceived AI as helpful in reducing human error and supporting rapid data processing, they also raised concerns about its limited integration with current medical record systems, thereby threatening workflow continuity.^47^

As a decision support tool, students recognised AI's ability to process large datasets efficiently and to enhance diagnostic accuracy.^8^, ^19^ Despite these advantages, concerns remained regarding AI's ability to fully grasp the nuances of clinical decision‐making. One student argued that “medicine is as much an art as a science”^43^ and cautioned that AI may underappreciate the complexity of clinical decisions shaped by subtle observations and patient relationships. As such, students emphasised that AI should remain a tool alongside clinical judgement and decision‐making processes.^57^ This balance was considered particularly important in specialties that rely heavily on data interpretation, such as radiology, where over‐reliance on AI could undermine the expertise of medical professionals.^55^

Beyond decision making, students raised concerns about AI's impact on the doctor‐patient relationship. Although current AI tools lack the capacity to replicate trust, empathy and respect, which remain integral to any doctor‐patient relationship,^43^ many feared that widespread adoption of AI in diagnostics and treatment could de‐personalise health care interactions.^57^ However, others saw a complementary role with AI tools freeing up clinicians' time to focus on human connection.^52^ These second‐order constructs highlight the tension between operational efficiency and humanistic care and prompt students to advocate a hybrid model that preserves clinical autonomy.

#### 3.3.2 AI integrity

Synthesised second‐order constructs included ethical issues, trust and accountability and inequity. Participants identified ethical integrity as a central concern in AI integration.^56^ One student warned that “major ethical and legal issues will arise if (and when) mistakes are made using AI as a guide”.^57^

Transparency and accountability in AI integration were consistently emphasised. Students expressed concerns with privacy, confidentiality and the “black box” nature of many AI systems.^8^, ^21^ Particularly, participants expressed scepticism about how data are collected, stored and used.^24^ Their concerns about data governance also extended to broader issues of responsibility and accountability for AI‐driven decisions, with participants questioning who is liable when AI systems make erroneous or biased decisions.^8^ To mitigate these risks, participants emphasised the need for “transparent and rigorous validation of these tools to ensure their safety and reliability in clinical settings”,^8^ though some admitted limited awareness of existing regulatory frameworks.^23^

Concerns about inequity in AI encompass both algorithmic biases in decision‐making processes and disparities in accessing these technologies. Students highlighted that if AI systems are trained on unrepresentative data, they risk reflecting the biases present and perpetuating existing health care inequalities.^52^ These biases may cause AI to perform differently on diverse patient populations and result in flawed decisions that directly harm patient health.^8^, ^19^, ^44^ However, some participants suggested AI could reduce human biases in clinical encounters by removing subjective assumptions based on appearance.^49^

These concerns on exacerbating disparities are more pronounced in low‐ and middle‐income countries, where inadequate technological infrastructure, such as limited internet access, could hinder AI integration.^19^, ^44^ Without a robust technological foundation, students and faculty members fear that AI advancements in wealthier regions may further widen global health care disparities, with one participant commenting that “AI [is] for just the rich”.^20^

#### 3.3.3 Educational implications and preparedness

Given AI's increasing role in clinical practice, students and faculty members highlighted that AI education in medical curricula is essential preparation for the future workforce.^56^ They emphasised the need for both technical literacy and critical evaluation skills and expressed strong interest in learning about AI ethics.^23^, ^44^ However, in regions with limited financial resources and technological infrastructure, such as in Nepal, Palestine, Nigeria and Morocco, participants questioned the relevance of learning AI in health care.^19^, ^20^, ^22^, ^44^

Approaches to integrating AI into medical curricula varied significantly. On the one hand, some universities in high‐income countries have incorporated AI topics into curricula and assessment to equip students with knowledge in AI diagnostics, treatment planning and patient management.^8^, ^54^ On the other hand, other students from high‐income countries reported minimal or no exposure to AI.^56^ In response, students have advocated for more structured approaches and hands‐on training in clinical settings.^58^ Recognising the interdisciplinary nature of AI, students also advocated for active collaboration with AI engineers, health care providers and patient experts during their learning.^23^

Three main perceived barriers hinder effective AI integration into medical curricula: poor knowledge of AI, low priority for AI education and overwhelming curricula. Many participants acknowledged a significant gap in their understanding of AI, stemming from both personal perceptions and a lack of AI and computer science teaching in pre‐medical and medical curricula.^23^, ^48^ Misconceptions about AI and its applications in health care also persist amongst faculty members, with one stating that “AI is just calculations on a larger scale”.^19^

This knowledge gap is further compounded by the perception that AI education is unimportant. Traditional medical curricula prioritise fundamental medical sciences and clinical care, and often relegate emerging technologies to a supplementary role. Some participants questioned AI's relevance, with competing priorities, not understanding personal relevance, and limited current visibility in clinical practice further diminishing its curricula priority.^23^, ^24^ A student from Pakistan stated “none of the medical colleges [is] working on artificial intelligence”,^52^ and a faculty member from Nepal admitted they had “no idea about where exactly in medical education […] AI stands”.^19^ Looking ahead, one student suggested that AI education would be more appropriate and important to learn during residency rather than medical school.^23^

Many students felt overwhelmed by their current medical curriculum and viewed the addition of AI education as a burden rather than a benefit.^22^, ^24^, ^56^ Students also expressed anxiety that “[learning AI] is simply not our field and expertise and it stresses [us] out”.^56^ These sentiments reflect students' apprehension about AI integration into medical curricula and uncertainty about their ability to adapt to evolving medical technologies without sufficient institutional support.

#### 3.3.4 Future workforce

The integration of AI into health care practice is perceived as both a threat for job security and an opportunity for professional growth. Although some participants fear that AI could replace roles traditionally occupied by humans,^23^, ^55^ others perceive AI as a catalyst for transforming clinical roles.^52^, ^57^

Job security concerns were particularly evident in relation to certain specialities. Most students agreed that specialties heavily reliant on data interpretation, such as radiology, histopathology and dermatology, would be most affected.^22^, ^50^, ^56^, ^57^ At the same time, students recognised that AI proficiency may become a distinguishing skill, with some believing that “doctors who can use AI will replace doctors who can't”.^57^

AI integration into clinical practice also evoked strong emotional responses among students. Although some students were energised by the prospect of working with emerging technologies,^23^, ^57^ others were uncertain and apprehensive. They admitted to feeling overwhelmed by its complexity,^24^ as well as confused and concerned about their future roles.^20^, ^57^

### 3.4 Tensions and alignments between third‐order constructs

**FIGURE 2:**
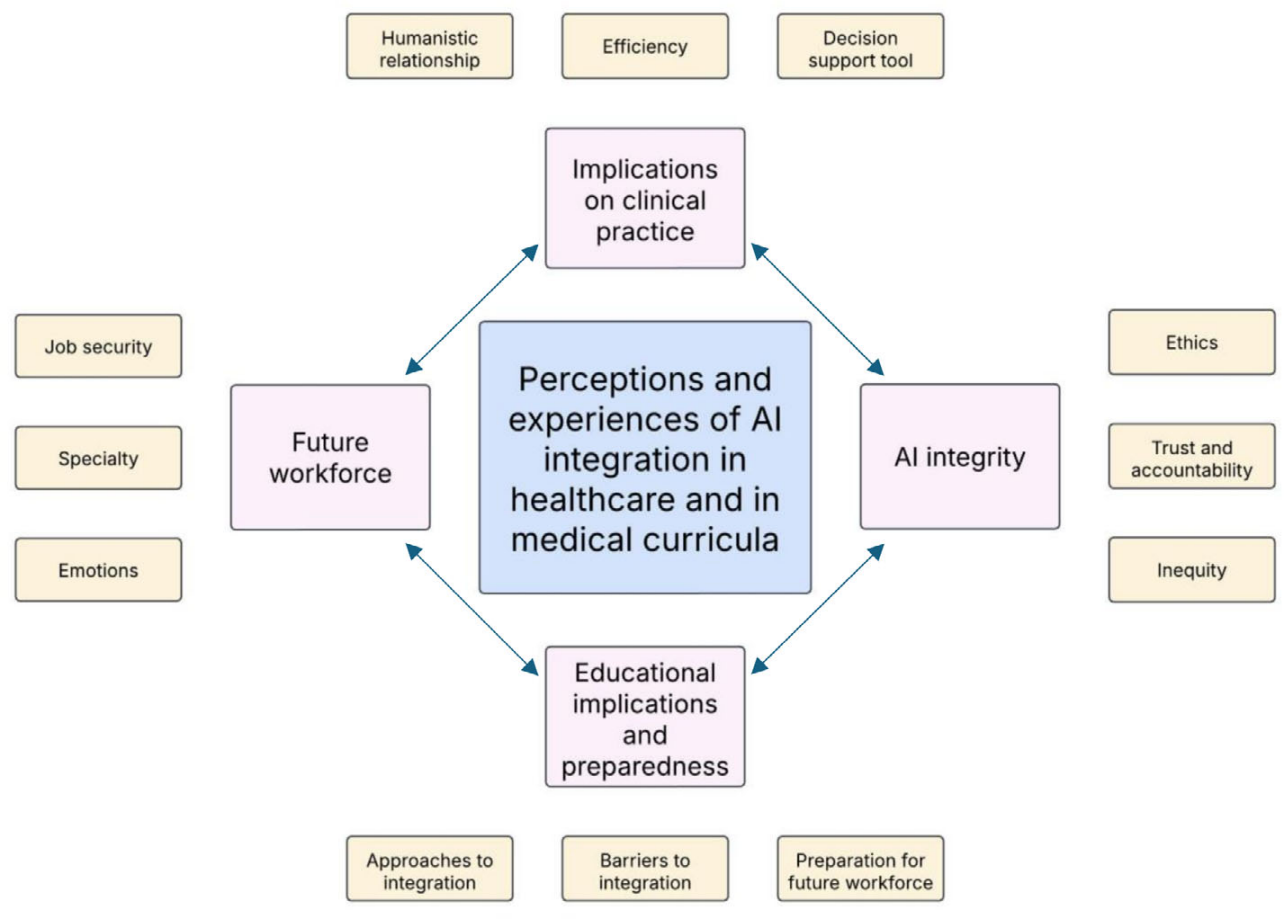
Second‐ and third‐order constructs of students' and faculty members' perceptions of AI integration in health care and in medical education. Yellow = second‐order constructs; pink = third‐order constructs. [Color figure can be viewed at wileyonlinelibrary.com]

Guided by Noblit and Hare's line‐of‐argument synthesis and the eMERGe guidelines,^29^, ^33^ we developed an interpretive representation to illustrate how third‐order constructs intersect (Figure 2). Through reciprocal and refutational translation, we identified links across the different constructs. Rather than treating constructs in isolation, this synthesis highlights how AI perceptions emerge from interconnected experiences in ethics, educational preparedness and evolving clinical roles.

The first linkage, **Implications of clinical practice** and **AI integrity,** reflects participants' acknowledgement of the tension between AI's potential to improve diagnostics and its ethical challenges, including confidentiality, bias and inequity. These insights highlight the need for strict ethical standards in the development and integration of AI in clinical settings.

The reciprocal relationship between **AI integrity** and **educational implications and preparedness** highlights participants' views that there is insufficient consideration and integration of these two constructs. Although students expressed a strong interest in learning about the technical, legal and ethical implications of AI, current curricula do not yet equip them adequately and faculty members feel underskilled to deliver such content. This mismatch in students' learning needs, faculty members' expertise and lack of curricular content risks students entering the workforce unprepared to navigate AI's challenges and potentially compromise patient safety.

The connection between **educational implications and preparedness**, and **future workforce** demonstrates participants' beliefs that curricular reforms need to anticipate evolving roles and responsibilities. Practical teaching methods, such as simulations, hackathons and workshops, were identified as key to equipping students with creative and collaborative problem‐solving skills. In parallel, interdisciplinary approaches that integrate data science and medicine were also recommended to strengthen cross‐sector collaboration and AI fluency.

The linkage between **future workforce** and **implications of clinical practice** highlights the need for ongoing re‐evaluation of clinical duties and professional identities. Although AI is seen to enhance diagnostics and efficiency, participants are concerned about doctors becoming passive overseers, losing professional autonomy and diminishing humanistic elements in care. Moving forward, participants emphasised the need to navigate a balance between leveraging these AI tools whilst maintaining patient‐centered care.

### 3.5 Emotional and cognitive dimensions of AI integration ‐ theoretical frameworks

Both Oberg's Culture Shock Model and Mezirow's Transformational Learning Theory offer a conceptual bridge between the reviews' findings and deeper interpretive insights.^59^, ^60^ The frameworks provide insight into how participants emotionally and cognitively navigate AI integration in health care practice and medical curricula.

Oberg's four‐stage model—Honeymoon, Frustration, Adjustment and Acceptance—helps explain how individuals emotionally respond to unfamiliar environments.^59^ Applied in the context of AI integration in health care practice and in medical education, it captures participants' evolving emotional transitions and maps closely onto our review's findings. In the Honeymoon phase, students and faculty show optimism about AI's promise to improve clinical decision‐making and operational efficiency,^58^ and express eagerness to engage with AI content in curricula.^57^ Frustration follows, as concerns emerge about algorithmic bias, job displacement and overcrowded curricula.^23^ In the Adjustment phase, participants describe gradual engagement with ethical AI use and curriculum reforms,^8^, ^19^ though progress varies by institutional resources. The Acceptance stage reflects appreciation of AI's value in augmenting rather than replacing human judgement.^52^, ^56^

Although the Culture Shock Model captures emotional trajectories, Transformational Learning Theory focuses on cognitive change.^60^ It postulates that disruptive events can prompt reflection and re‐evaluation of prior assumptions, thereby leading to deeper learning. AI disrupts clinical and educational norms, prompting participants to reconsider professional roles (Future Workforce) and ethical responsibilities (AI Integrity). Participants move from viewing AI as mere data tools^21^ to appreciating its broader diagnostic and ethical implications.^52^ Disorienting dilemmas—such as AI contradicting clinical judgement—foster critical reflection and identity shifts.^23^, ^43^ Transformational learning also informs curricular recommendations and calls for interactive approaches like PBL, simulation and case‐based learning to foster reflective practice.^24^, ^48^ These methods align with the theory's emphasis on engaging students in reassessing assumptions in light of new realities.

## 4 DISCUSSION

### 4.1 Strengths and limitations of the review

Meta‐ethnography is a robust methodology for synthesising qualitative studies.^39^ The systematic search process documented through the PRISMA diagram and the critical appraisal of study quality using the CASP framework have enhanced the review's comprehensiveness and rigour. With 26 included studies, this review represents a medium‐scale synthesis, maintaining balance between depth and breadth. Campbell et al^39^ cautioned that larger reviews (>40 studies) risk sacrificing depth for breadth.

However, several limitations may have affected the synthesis. Firstly, the interpretive nature of meta‐ethnography can introduce variability in reproducibility and interpretation.^39^ Although reflexivity and a balanced insider‐outsider perspective were maintained to mitigate bias, personal and academic experiences may still have influenced interpretations. Potential biases may arise from overlooking minority viewpoints and challenges in integrating diverse educational and cultural contexts. The restriction of the inclusion criteria to articles published in English‐language and in peer‐reviewed journals may have excluded insights from students and faculty members who documented their experiences in other languages or published in another format. Furthermore, the definitions of “AI” were inconsistently reported across studies, and few studies specified whether participants were provided with the definition; this may have affected comparability of perspectives and experiences.

Although this review has included papers from more than 48 countries, the predominant perspectives are from developed regions 3: North America (42%), Western Europe (35%) and Oceania (8%). This geographical imbalance may have influenced the synthesis, particularly around issues of educational and workforce infrastructures. It could also limit the applicability and transferability of the findings across diverse health care systems and cultural contexts. Given the diversity within individual countries, further context‐ and culture‐specific analyses are needed.

### 4.2 Implications for medical education

A comprehensive revision of curricula content, teaching methodologies and assessment strategies is essential to successfully integrate AI topics into medical education. Students have expressed interest in learning both the technical and ethical aspects of AI.^23^ Studies from this review suggest that curricula content should include computer science, data analytics and machine learning.^44^, ^52^ These findings align with existing literature, including Gordon *et al* ^25^ and Paranjape *et al*,^16^ who emphasised the importance of improving technical competencies in students. To integrate ethics into AI education, Weidener and Fischer^61^ proposed using the principles of medical ethics—autonomy, beneficence, nonmaleficence and justice—as a teaching framework. Barriers to AI integration in medical curricula also need to be addressed. Given an already packed curriculum, logistical challenges of allocating time and space for AI medical education teaching must be considered. Existing current curricula should be reviewed to identify modifiable or replaceable content.^62^

Curricula changes should be accompanied by changes in teaching methodologies. Integrating problem‐based and case‐based learning could further develop students' analytical and reasoning skills. For instance, learning cases can specifically focus on implications of AI decision‐making and ethical scenarios in clinical settings, aligning closely with students' interests and learning needs. These approaches are also consistent with recommendations from existing literature on AI and medical education.^1^, ^7^ Additionally, incorporating hands‐on training with AI tools and simulations could significantly enhance student confidence and familiarity with these technologies.^26^

Interdisciplinary learning can facilitate a more holistic understanding of AI's multi‐faceted impact on health care.^19^, ^44^ This approach requires data scientists, practicing doctors and medical educators to collaboratively identify learning needs and tailor teaching topics in computer science, data analytics and engineering.^54^ Despite potential logistical, financial and institutional barriers, institutions should consider exploring innovative teaching strategies, such as joint workshops, co‐teaching arrangements and integrated projects.^21^

Although this meta‐ethnography primarily focused on AI integration in medical curricula, existing literature highlighted the need for assessment reforms to ensure AI competencies are evaluated in both formative and summative assessments.^16^, ^63^ Assessments should move beyond testing students' knowledge and skills and instead measure their abilities to critically appraise AI technologies within real‐world health care contexts.^64^ For instance, case studies on ethical dilemmas and technology failures can enhance students' critical analysis skills,^65^ and data interpretation exercises can help students evaluate the accuracy and clinical relevance of AI‐generated outputs.^64^

### 4.3 Implications for future research

The review identified a gap in understanding faculty members' perceptions and experiences with AI in health care and medical curricula, with only 75 faculty members represented across 3 studies.^21^, ^54^, ^58^ Further research is needed to validate these findings and explore appropriate interventions.

Additionally, the synthesis lacked insight into student‐faculty interactions and how these dynamics influence individual perceptions of AI integration in health care and medical curricula. This is particularly relevant in the context of the rapid technological advancements, where newer generations are more attuned to emerging technologies.^66^ Existing research has already shown that medical students know more about AI than senior doctors.^67^ In educational systems characterised by pronounced hierarchical structures and significant power distances between students and faculty members, understanding these interactions is key to assessing how AI technologies are accepted and integrated. It can also help develop tailored strategies that address the diverse needs and capabilities of both groups.^31^, ^67^

Future studies should also evaluate how variations in AI teaching, such as curricula content and teaching methodologies, affect the experiences of both students and faculty members. Specifically, further research could identify best practices and barriers in interdisciplinary AI education. To translate these findings into practice, establishing a collaborative international consortium including all stakeholder voices would be beneficial in developing meaningful and sustainable AI educational strategies.

AI integration in medical curricula needs to be inclusive and sensitive to diverse cultural attitudes. Utilising post‐colonialism frameworks can help assess whether AI curricula and teaching methods are inadvertently perpetuating colonial legacies by enforcing standardised educational norms.^68^ This analysis is particularly important in non‐Western contexts, where globalising educational innovations risk overlooking local cultural nuances.^68^ Addressing these issues is essential, not only for tailoring AI education to local needs but also for mitigating ethical dilemmas and reducing resistance to AI adoption among students and faculty members. Akin to the integration of AI in health care, its integration in medical curricula also needs to enhance global educational practices without exacerbating existing inequalities.

This review, consistent with findings from Gordon *et al's* scoping review,^25^ found no qualitative studies examining the long‐term impact of AI in medical education or longitudinal studies assessing changes in perception over time. This gap likely reflects the early stage of AI integration in medical curricula; therefore, future longitudinal research would be valuable for tracking changes in perceptions and understanding the long‐term impact of AI integration.

## 5 CONCLUSION

This review critically evaluated the diverse perceptions and experiences of medical students and faculty members globally on AI integration in health care and in medical curricula. Through a meta‐ethnographic synthesis of qualitative studies, it highlighted how they perceive the transformative impacts of AI in health care and the various opportunities and challenges in medical curricula.

This review lays the groundwork for future research and policy discussions. Moving forward, collaborative and forward‐thinking discussions among educators, policymakers and health care professionals are essential. A strategic and inclusive approach would ensure that AI is integrated into medical education and health care delivery in a way that is ethical, effective and equitable.

## AUTHOR CONTRIBUTIONS

SCCC developed the research question and methodology, and drafted the first version of the manuscript. SCCC, HY and RP all contributed to data collection, analysis, and synthesis. All authors contributed to manuscript revision and gave final approval to this submitted paper. All authors agreed to be accountable for all aspects of the work.

## CONFLICT OF INTEREST STATEMENT

The authors declare no conflicts of interest.

## ETHICS STATEMENT

Not applicable.

## Supporting information


**Appendix S1:** Search terms and strategies.

## Data Availability

This study is a meta‐ethnographic synthesis of existing qualitative research. No new primary data were generated during this study. All data are derived from previously published articles, which are cited in the manuscript and publicly available.

## Footnotes

^1^
The total number of students involved in this review is estimated from the 26 included studies. In cases where mixed‐methods studies did not specify the number of participants in the qualitative component, all study participants were included. Additionally, due to anonymity constraints in the studies, it is not possible to determine whether there are duplicate participants across different studies.

^2^
At least 48 countries are represented in this review. One paper^44^ reported findings from participants from 48 countries, but did not specify which countries are included.

^3^
The regional distribution of studies in this review reflects their origins. Studies spanning multiple regions are counted separately for each region involved.
