# Supplementary material for: Medical students' and faculty members' perceptions and experiences of AI integration in health care practice and in medical curricula: A meta‐ethnographic review
Source: Med Educ. 2025 Nov 5;60(5):492–504. doi: 10.1111/medu.70071 (PMC13067092; doi:10.1111/medu.70071)
Supplement: Supplementary file 1 — Appendix S1: Search terms and strategies. [file MEDU-60-492-s001.docx]

Appendix 1: Search terms and strategies

| Database | Search strategies |
| --- | --- |
| Web of science | medical NEAR/0 (school* or education or teacher* or lecturer* or professor* or student* or curricul* or facult* or undergrad*) |
|  | *AND* |
|  | "Artificial intelligence" or AI or "machine learning" or "Natural language processing" or "Algorithmic Processing" or "Algorithmic learning" or chatbot* or Digital chatbox or ChatGPT |
|  | *AND* |
|  | "Focus Group*" or Interview* or Qualitative or Transcript* or "mixed method*" or Questionnaire* or Survey* |
|  | *AND* |
|  | Evaluat* or Perception* or Attitude* or Experience* or Challenge* or Barrier* or Acceptance or View or views or Feedback or Reaction* or Opinion* or Facilitator* |
|  | |
| ERIC  (through EBSCO host) | TI (medical N0 (school* or education or teacher* or lecturer* or professor* or student* or curricul* or facult* or undergrad*)) OR AB (medical N0 (school* or education or teacher* or lecturer* or professor* or student* or curricul* or facult* or undergrad*)) |
|  | *AND* |
|  | TI ("Artificial Intelligence" or "AI" or "machine learning" or "Natural language processing" or "algorithmic processing" or "Algorithmic learning" or "digital chatbox" or chatbot* or chatgpt) OR AB ("Artificial Intelligence" or "AI" or "machine learning" or "Natural language processing" or "algorithmic processing" or "Algorithmic learning" or "digital chatbox" or chatbot* or chatgpt) |
|  | *AND* |
|  | TI (interview* or "focus group*" or "mixed method*” or questionnaire* or qualitative or survey* or transcript* ) OR AB ( interview* or "focus group*" or "mixed method*” or questionnaire* or qualitative or survey* or transcript* ) |
|  | *AND* |
|  | TI (evaluat* or perception* or attitude* or experience* or challenge* or barrier* or facilitator* or acceptance or view or views or feedback or reaction* or opinion*) OR AB (evaluat* or perception* or attitude* or experience* or challenge* or barrier* or facilitator* or acceptance or view or views or feedback or reaction* or opinion*) |
|  | |
| PsycINFO  EMBASE  Medline  (through OVID) | (Medical adj1 (school* or education* or teacher* or lecturer* or professor* or student* or curricul* or facult* or undergrad*)).ti,ab |
|  | *AND* |
|  | ("Artificial Intelligence" or "AI" or "machine learning" or "Natural language processing" or “algorithmic processing” or “Algorithmic learning” or “digital chatbox” or chatbot* or chatgpt).ti,ab |
|  | *AND* |
|  | (interview* or "focus group*" or "mixed method*" or questionnaire* or qualitative or survey* or transcript*).ti,ab. |
|  | *AND* |
|  | (evaluat* or perception* or attitude* or experience* or challenge* or barrier* or facilitator* or acceptance or view or views or feedback or reaction* or opinion*).ti,ab. |
